# Supplementary material for: Negative longitudinal magnetoresistance in gallium arsenide quantum wells
Source: Nat Commun. 2019 Jan 17;10:287. doi: 10.1038/s41467-018-08199-2 (PMC6336836; doi:10.1038/s41467-018-08199-2)
Supplement: Supplementary file 1 — Supplementary Information [file 41467_2018_8199_MOESM1_ESM.pdf]

Supplementary Information

Negative longitudinal magnetoresistance in gallium arsenide quantum wells

Jing Xu *et al*

Parameters / Samples	$R_0(\Omega)$ at 300K	n ($10^{10}cm^{-2}$) at 3K	T_p (K)	T_M (K)	Maximal NLMR	N_0 ($10^{12}cm^{-2}$)	E_A (meV)
Sample W1a	3451	8.16	150.5	180	-4.16%	3.45	38.8
Sample W1b	2420	8.72	145	165	-6.14%	3.05	34.1
Sample W1c	1366	8.83	144	165	-7.68%	2.25	31.9
Sample W2	4002	7.91	154	190	-3.85%	2.23	35.3

Supplementary Table 1. Summary of parameters for the measured samples. T_p is the temperature at which the R_0 (T) curve has a peak (see Fig.2a); T_M is the temperature at which maximal NLMR occurs for that sample; N_0 and E_A are derived from the fit of $n(T)$ curve at high temperatures using $n = N_0\exp(-E_A/k_BT)$ (see Fig.2b).

a

b

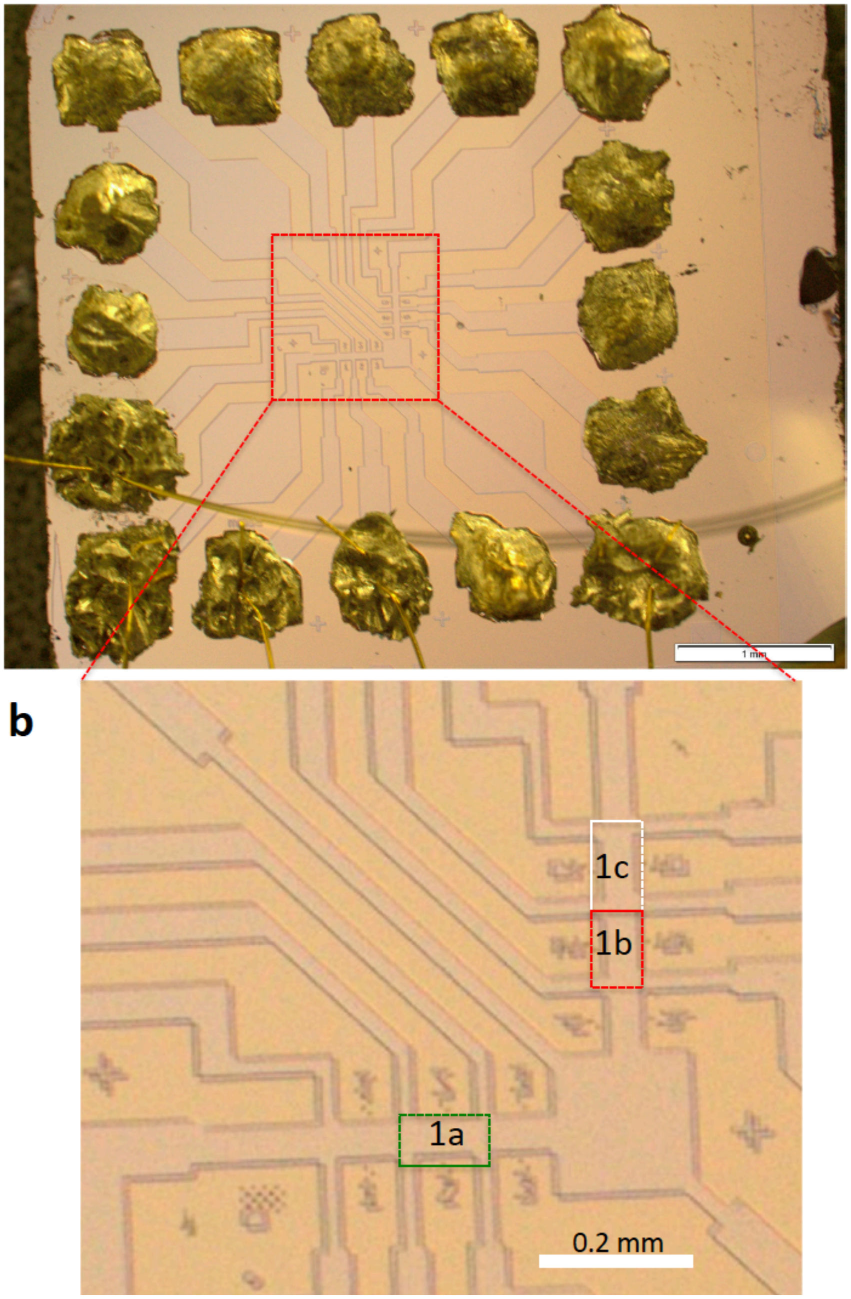

Supplementary Figure 1 | Photograph of the GaAs quantum well wafer with fabricated Hall bar samples. **a**, The wafer W1 with electrical contacts. Dashed rectangles highlight the three measured Hall bars. Indium was used to make the Ohmic contacts. Gold wires of 50 μm in diameter were used for electrical connections. **b**, Expanded view showing the locations of Samples W1a, W1b and W1c.

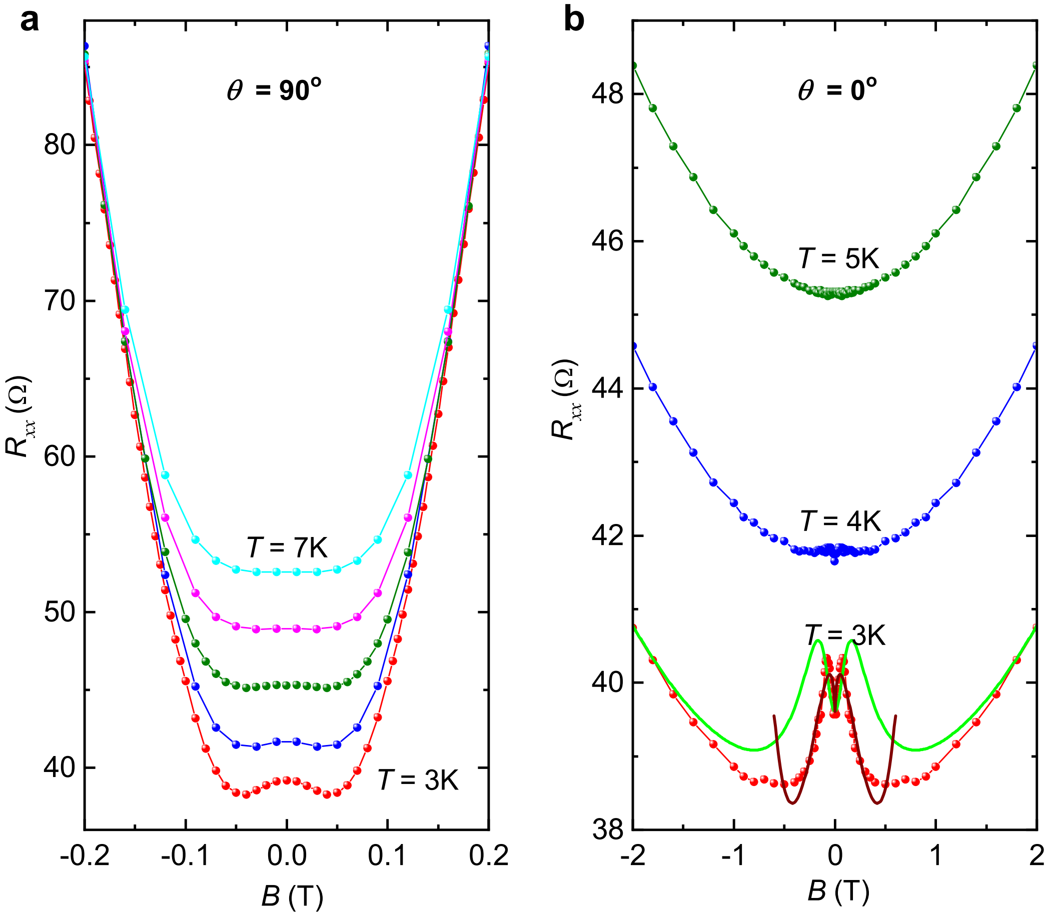

Supplementary Figure 2 | Negative magnetoresistances in Sample W1b at liquid helium temperatures. **a**, at **B**⊥ **I** and **b**, at **B**//**I**. The green and purple curves in (**b**) are derived from Eq.2 and Eq.3, respectively.

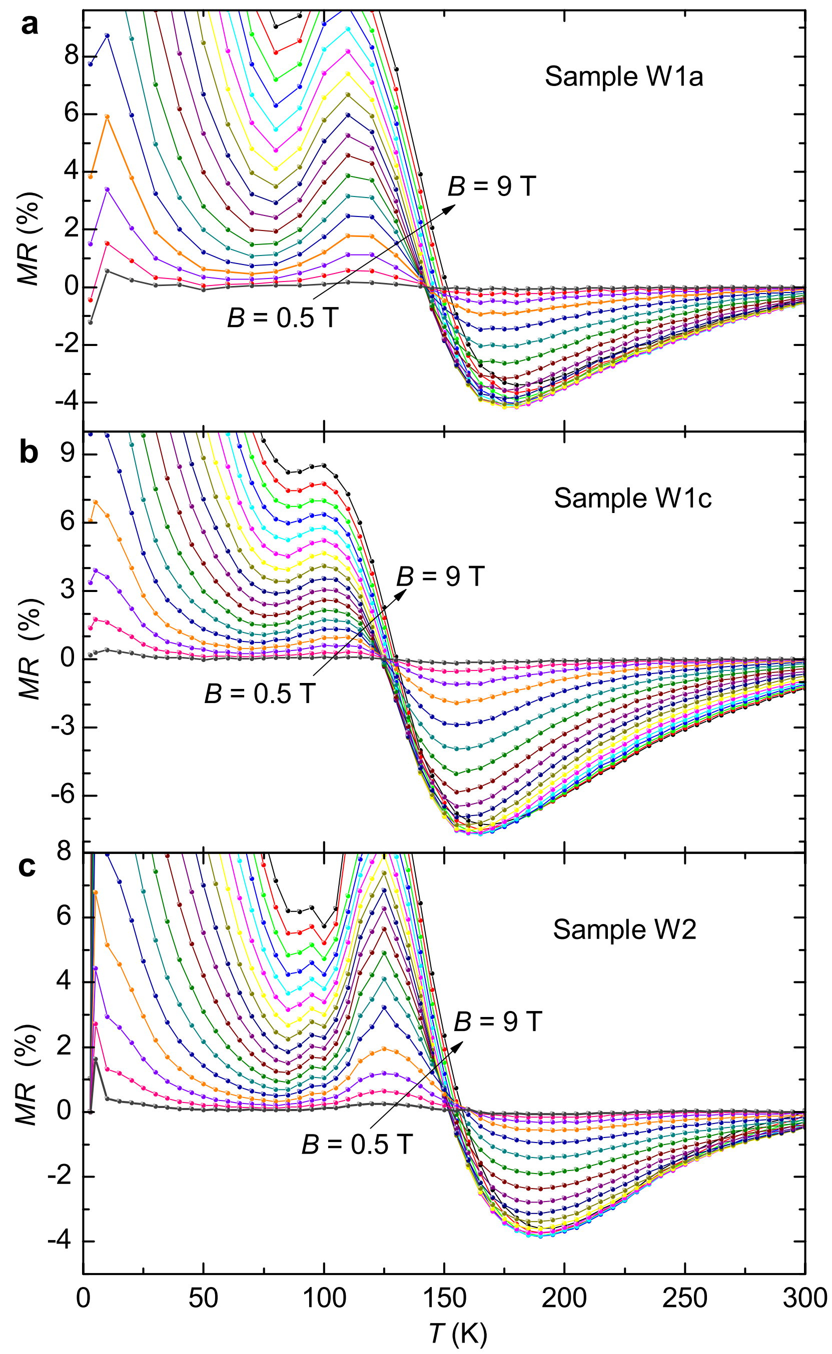

Supplementary Figure 3 | Temperature dependence of the longitudinal magnetoresistance. **a**, Sample W1a, **b**, Sample W1c, and **c**, Sample W2.

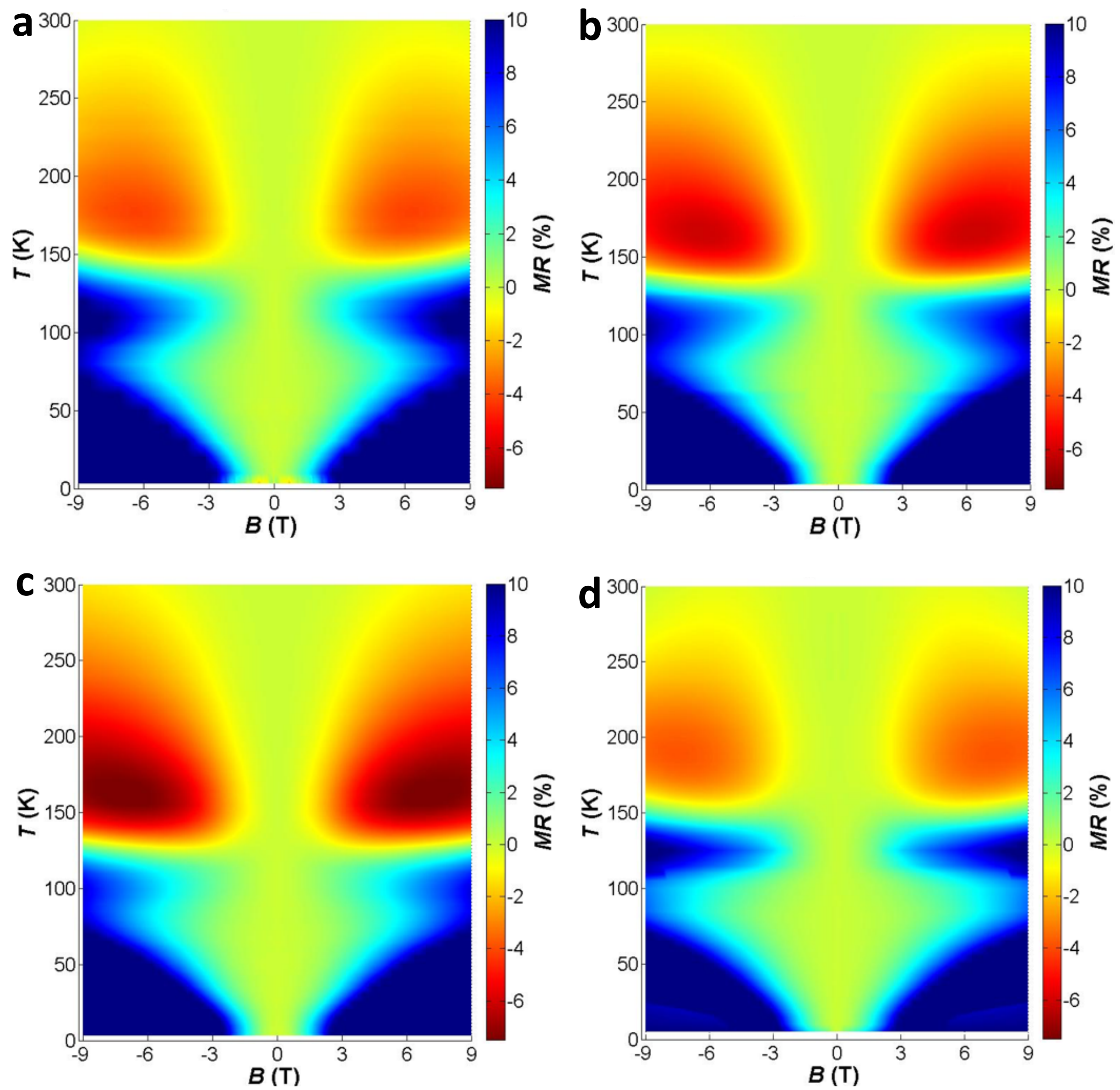

Supplementary Figure 4 | Color maps for the longitudinal magnetoresistances in all four samples. **a**, Sample W1a, **b**, Sample W1b, **c**, and Sample W1c, and (**d**) Sample W2.

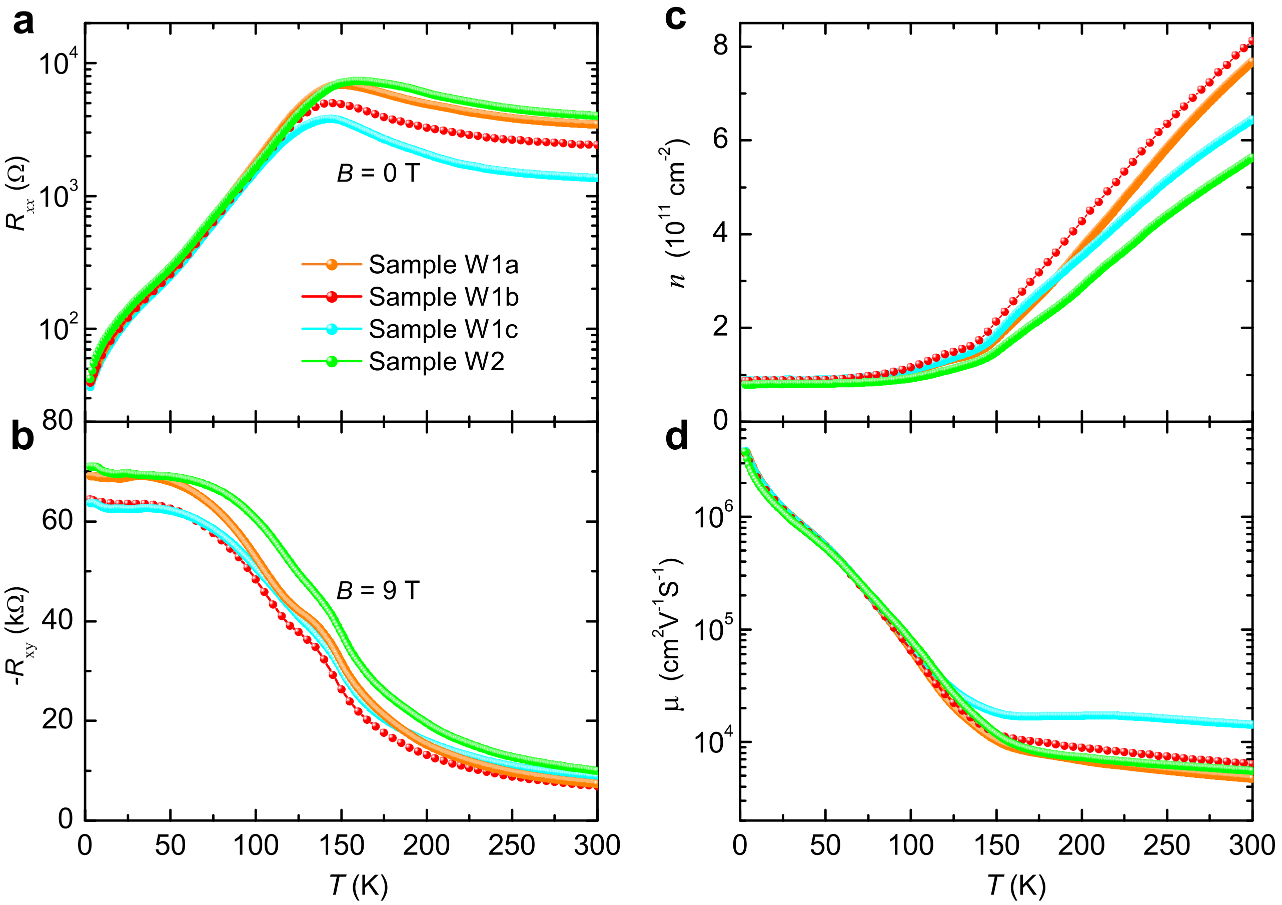

Supplementary Figure 5 | Basic characterizations of the measured samples. a, **b**, **c**, and **d** show the temperature dependence of the zero-field resistance, the Hall resistance at B = 9 T, the electron density and mobility respectively. The electron densities n in (**c**) is calculated from the Hall resistances R_{xy} in (**b**) through the relationship $R_{xy} = B/ne$. The mobility μ in (**d**) is derived from the zero-field resistivity $\rho_0 = R_{xx}(0\text{T})L_y/L_x$ in (**a**) and the electron density n in (**c**) through the relationship $\rho_0 = 1/ne\mu$

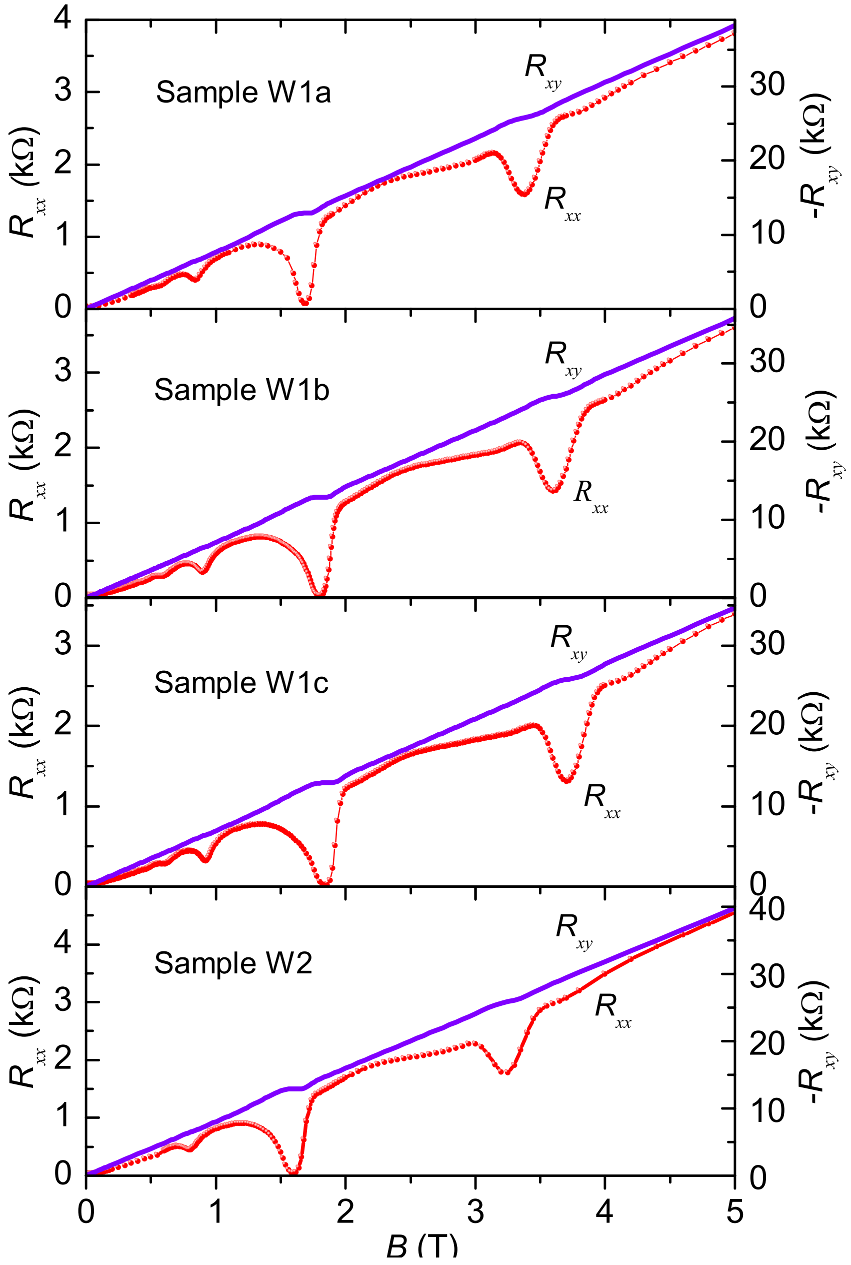

Supplementary Figure 6│ Shubnikov de Haas quantum oscillations and quantum Hall effect. Data were taken at $T = 3$ K and in magnetic fields perpendicular to the quantum well plane. For clarity data only up to $B = 5$ T are presented in the figure.

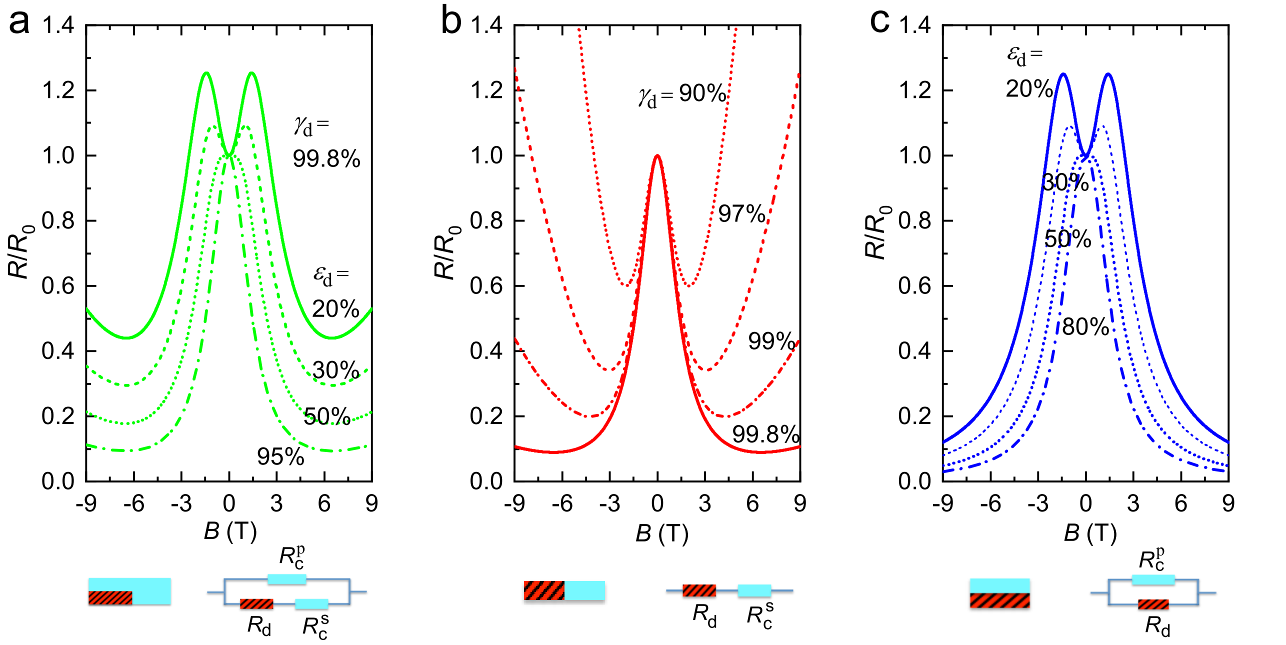

Supplementary Figure 7 | Numerical results from the phenomenological model. These results show characteristic features of NLMRs in various scenarios of disorder distribution. **a,** the general case that requires all three resistors (R_d, R_c^p and R_c^s) in the equivalent circuit, which describes samples with dispersed distribution of the disordered areas. **b,** a simpler scenario that only requires an equivalent circuit of R_d and R_c^s in series, which represents samples with connected disordered areas across the sample in the direction perpendicular to the current. **c,** a simpler scenario that can be described with R_d and R_c^p in parallel, which represents samples with connected disordered areas across the sample in the direction parallel to the current. The schematics and corresponding equivalent circuits are given at the bottom of each panel. The $R(B)/R_0$ curves in (**a**) were obtained by using Eq.2 with all five variables, i.e., $R(B)/R_0 = \{\varepsilon_d/[\gamma_d/(1+\alpha B^2) + (1-\gamma_d)(1+\beta^s B^2)] + (1-\varepsilon_d)/(1+\beta^p B^2)\}^{-1}$. The results in (**b**) were derived using reduced form of Eq.2 with $\varepsilon_d = 1$, i.e., $R(B)/R_0 = [\gamma_d/(1+\alpha B^2) + (1-\gamma_d)(1+\beta^s B^2)]$. The $R(B)/R_0$ curves in (**c**) were calculated using reduced form of Eq.2 with $\gamma_d = 1$, i.e., $R(B)/R_0 = [\varepsilon_d(1+\alpha B^2) + (1-\varepsilon_d)/(1+\beta^p B^2)]^{-1}$. We used $\alpha = \beta^s = \beta^p = 0.5$ T^{-2} in all calculations and the values of ε_d and γ_d for each case are given inside the corresponding panels. These results provide guidance in choosing a reduced form of Eq.2 for analyzing the experimental data, leading to more reliable outcomes by reducing the number of variables and also uncovering the disorder distribution in the sample. For example, the experimental $R(B)$ curves at $T > 138$ K in Fig.1e follow the characteristic features of the NLMRs in (**b**). Thus, we used $R(B)/R_0 = [\gamma_d/(1+\alpha B^2) + (1-\gamma_d)(1+\beta^s B^2)]$ for the analysis (see text for the relevant discussion).

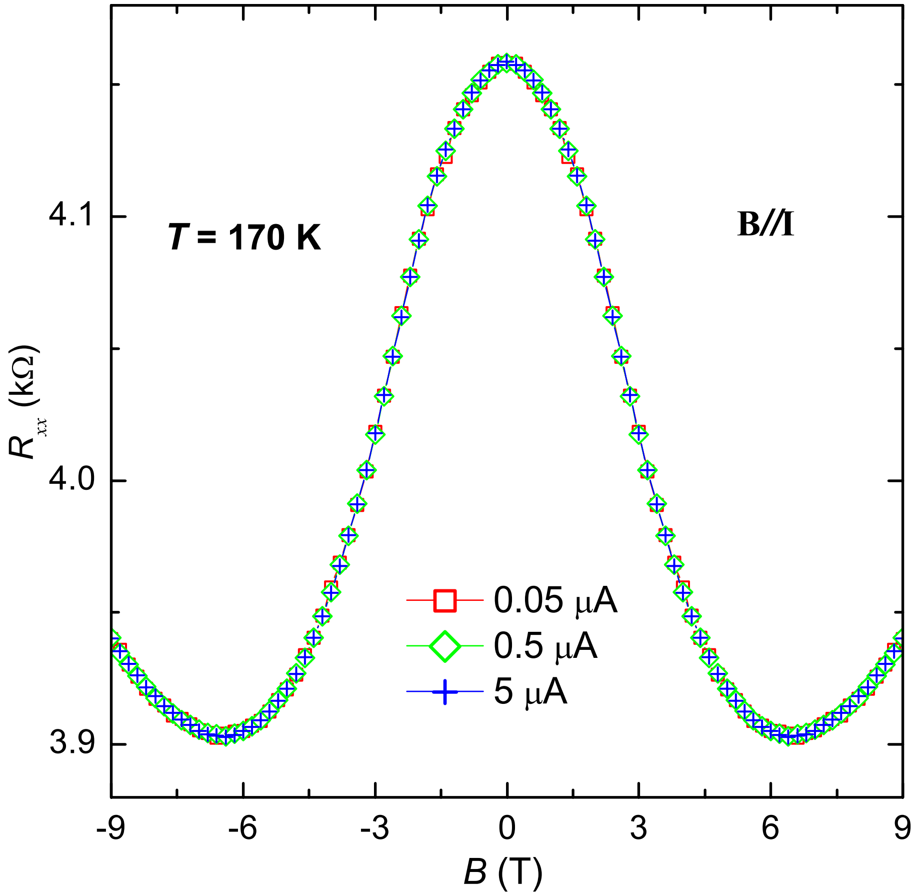

Supplementary Figure 8 | Current independence of the negative longitudinal magnetoresistance. Data were taken in Sample W1b at T = 170 K.

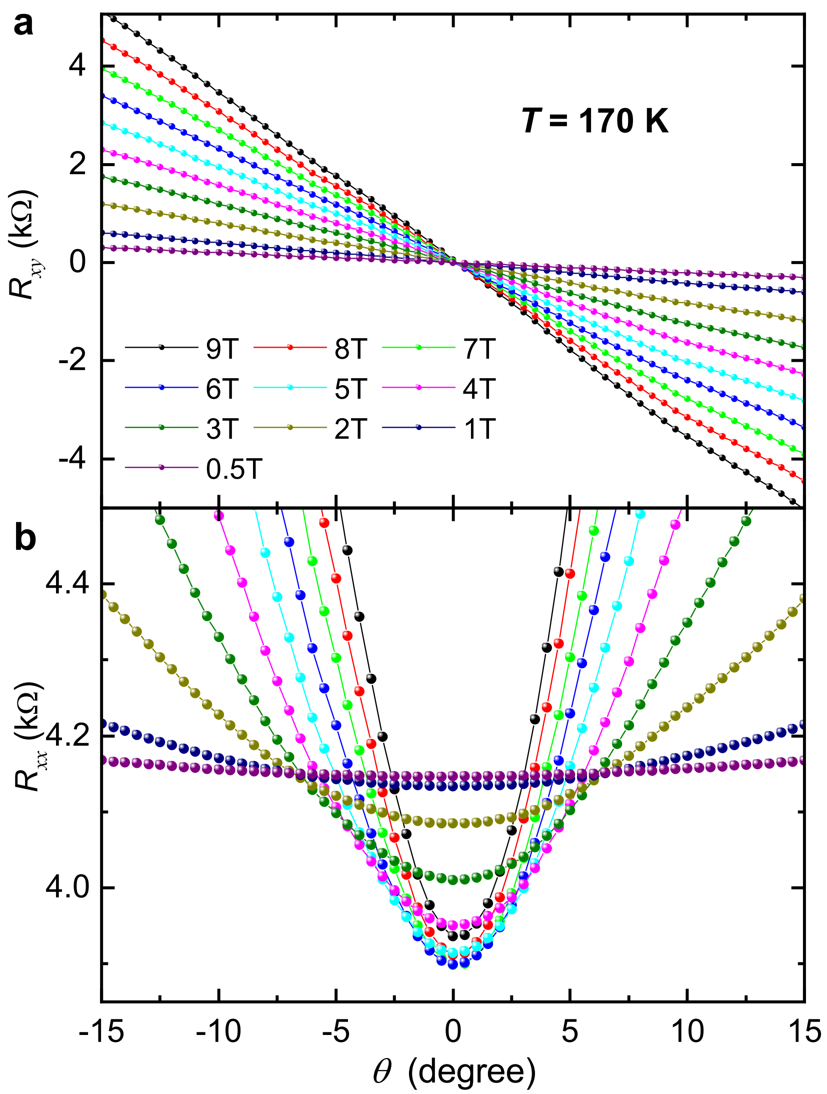

Supplementary Figure 9 | Experimental approach to define the angle between the magnetic field B and the current I . At **B**//**I**, R_{xy} is expected to be 0 and R_{xx} should be at minimum. To determine this orientation, we measured the angle dependence of R_{xy} and R_{xx} in Sample W1b at various magnetic fields, which are presented in (**a**) and (**b**), respectively. The angle at which R_{xy} is 0 and R_{xx} is at minimum is defined to be $\theta = 0°$.
